# Osimertinib-Centered Therapy Against Uncommon Epidermal Growth Factor Receptor-Mutated Non-Small-Cell Lung Cancer- A Mini Review

**DOI:** 10.3389/fonc.2022.834585

**Published:** 2022-04-14

**Authors:** Chengyang Song, Xueying Yang

**Affiliations:** The Department of Thoracic and Cardiovascular Surgery, The Fourth Affiliated Hospital of China Medical University, Shenyang, China

**Keywords:** EGFR targeted therapy, osimertinib-sensitive/resistant uncommon EGFR mutations, structural elucidation, individualized medication, non-small-cell lung cancer

## Abstract

Osimertinib is a third-generation, irreversible mutant epidermal growth factor receptor (EGFR) tyrosine kinase inhibitor that is approved by the U.S. Food and Drug Administration (FDA) and European Medicines Agency (EMA). Osimertinib is currently the first line drug recommended by National Comprehensive Cancer Network (NCCN) guidelines against lung cancer harboring the EGFR TKI-sensitive mutation and acquired EGFR T790M resistance mutation. Osimertinib demonstrated some efficacy in clinical trials and case reports in patients bearing certain uncommon EGFR mutations, but it is not active in patients with other mutations such as C797S. This mini-review presents the mechanisms underlying the variations in patient responses, discusses the use of osimertinib against non-small-cell lung carcinomas with uncommon EGFR mutations, and addresses the future prospects of osimertinib-centered therapy.

## Introduction

As one of main contributors to cancer-related mortality, lung cancer imposes huge economic, physical and psychological burdens to families and the health system. There is an estimated 2.2 million new cases of lung cancer and 1.79 million deaths from lung cancer each year worldwide. The cost for the diagnosis, surgery and chemo-/radiation therapy is approximately 40,000 USD per patient (1–3).

About 85% of all lung cancer cases are non-small-cell lung cancer (NSCLC) (1, 4). NSCLC is mainly treated by surgical resection and platinum-based chemotherapy. However, resistance to chemotherapy and recurrence is common, leading to a poor survival rate of patients with NSCLC. Therefore, more effective treatments for NSCLC are required.

Aberrant epidermal growth factor receptor (EGFR) activity, one of the most common phenomena in NSCLC, drives the uncontrollable growth of lung cancer cells. EGFR contains an extracellular ligand-binding domain, a transmembrane domain, a cytoplasmic tyrosine kinase (TK) domain and a carboxy terminal domain (5). In lung cancer, the TK activity is frequently dysregulated by several oncogenic mechanisms, including EGFR gene mutation, EGFR gene copy number alternation and overexpression of EGFR protein (6). Gain-of-function mutations in EGFR in NSCLC cells results in constitutive activation of EGFR signaling, leading to apoptosis resistance and an exaggerated growth phenotype (6). The interaction between EGFR and the integrin signal pathway mobilizes cancer cells by activating matrix metalloproteinases and changing cell adhesion, ultimately causing metastasis (5). Therefore, EGFR is considered as a druggable target and a hotspot for the future rational design of new drugs for the treatment of NSCLC patients.

The development of tyrosine kinase inhibitors (TKIs) targeting EGFR carrying TKI-sensitive mutations has promoted the evolution of NSCLC therapy (6, 7). Gefitinib is one of the first generation TKIs and opened the door for precise targeted treatment for cancer patients (7). Although the first generation TKIs showed promising therapeutic effects, approximately 50–60% of tumors become resistant to TKIs through a mechanism involving acquired T790M mutation (8). The second generation TKIs afatinib and dacomitinib overcame the drug resistance problem, but as multi-target inhibitors, these TKIs act against wild-type EGFR, causing more side effects. In addition, their therapeutic dose against EGFR T790M exceeded the maximum tolerant dose, which reduced treatment adherence (9). Thus, the third generation TKIs were developed with enhanced selectivity and manageable toxic side effects. In 2019, osimertinib, a third-generation TKI, was indicated by the National Comprehensive Cancer Network (NCCN) Guidelines for NSCLC as the first line drug against NSCLC with TKI-sensitive EGFR and T790M mutations (10).

The common EGFR mutations in NSCLC include mutations in exons 19 and 21. Uncommon EGFR mutations are a group of heterogeneous remainders in exon 18 to 21 that comprise approximately 20% mutations of cancer-related EGFR mutations (11). These mutations are important contributors to cancer progression and relapse. This mini-review summarizes the efficacy of osimertinib in NSCLC patients with uncommon EGFR mutations and discusses the future directions for osimertinib-centered therapy.

## Osimertinib and Its Pharmacology

Osimertinib, initially called AZD9291 (12), is a potent and selective EGFR inhibitor and third generation TKI. Osimertinib is a member of the aminopyrimidine family, in which one of the amino hydrogens is replaced with a 2-methoxy-4-[2-(dimethylamino)ethyl](methyl)amino-5-acrylamidophenyl group. Osimertinib covalently binds to numerous kinds of EGFRs with mutations at C797 with no effects on wild-type EGFR (13, 14).

The potency of osimertinib is reflected by its pharmacological features. It takes 6 h for the chemical to reach the maximal plasma concentration (Cmax) upon oral administration and approximately 15 h to achieve steady state. It has a large volume of distribution (918 L at steady state). The liver cytochrome enzymes CYP3A4/5 metabolize the drug into AZD7550 and AZD5154 showing 10% of plasma activity at steady state. Osimertinib has an approximate 14.3 liter per hour clearance and a comparatively long half-life of 50 h. Food intake and proton pump inhibitors do not influence the exposure to the drug in healthy volunteers and NSCLC patients (15). Simultaneous administration of osimertinib and the CYP3A4 inducer itraconazole hardly influences the exposure to either osimertinib or its metabolite AZD5154 significantly in NSCLC patients. NSCLC patient exposure to osimertinib and AZD5154 drops upon osimertinib administration accompanying with the strong CYP3A4 inhibitor rifampicin intake. The two phenomena are causally related, and the effect of concomitant administration is reversible if rifampicin administration is terminated (16).

A population pharmacokinetics model supports a positive risk-benefit relationship of the 80 mg-per-day dose approved in the USA, EU and Japan across different population-based covariates (17). Compared with alternative therapeutic methods, osimertinib at the dose of 80 mg/day is associated with limited diarrhea, rash or abnormal electrocardiogram reading (18). Osimertinib has a large therapeutic window, considering that there was no maximum tolerant dose observed after testing the clinical activities at all doses (17). The pharmacokinetics of osimertinib is minimally affected by ethnicity-related genetic polymorphisms, age, gender and smoking status. Covariates affecting the pharmacokinetics of osimertinib do not dramatically influence its exposure to require dose adjustments (17).

## The Efficacy of Osimertinib in NSCLC Patients With Uncommon EGFR Mutations

A multicenter, single-arm, open-label, phase II study performed by a group of Korean clinicians, has been the only published clinical trial, to investigate the efficacy of osimertinib in NSCLC patients with uncommon EGFR mutants. The study enrolled 37 patients from 7 institutes in Korea; one patient later withdrew consent. All the enrolled patients were histologically diagnosed as metastatic or recurrent NSCLC with uncommon EGFR mutations. At the time of data cutoff, 22 cases of disease progression occurred, and 7 patients died. Approximately 50% of 36 patients showed a partial response (PR) and 39% of the cases maintained stable disease. The objective response rate (ORR) was 50% (18 of 36 patients; 95% confidential interval (CI), 33% to 67%), and the disease control rate (DCR) was 89% (32 of 36 patients; 95% CI, 78% to 100%). The median progression-free survival (PFS) reached 8.2 months (95% CI, 5.9 to 10.5). The percentages of patients who were free from progression were 64% (95% CI, 47% to 80%) at 6 months and 39% (95% CI, 22% to 56%) at 12 months. The 12-month survival rate was 86% (95% CI, 74% to 98%), and the 18-month survival rate was 56% (95% CI, 39% to 73%). A subset analysis of ORR and PFS was performed based on uncommon EGFR mutation type, including the G719X, L861Q, and S768I mutations. The ORR rates were 78% in patients with the L861Q mutation, 53% in patients with G719X and 38% for patients with S768I. The PFS rates were 15.2 months in patients harboring L861Q (95% CI, 1.3 to 29.1 months), followed by 8.2 months in patients with G719X mutation (95% CI, 6.2 to 10.2 months), and 12.3 months for patients with S768I mutation (95% CI, 0 to 28.8 months). The lesion size of four patients with compound uncommon EGFR mutations decreased; two of these patients harbored G719X and L861Q, and the other two harbored G719X and S768I.

Although studies on the NSCLC preclinical models are preliminary to some extent, they still support osimertinib as potent inhibitor of mutant EGFR as well as the latent efficacy in patients. A previous study conducted *in-vitro* phosphorylation assays on an engineered COS cell line expressing a panel of uncommon mutant EGFRs (G719A/C/S, L861Q, S768I and L747S) individually and in combination. The results demonstrated a potent inhibitory effect of osimertinib on mutant EGFR phosphorylation and the downstream signaling with IC50 values ranging from 4.5 to 40.7 nM (19). They study showed similar effects on the NSCLC patient–derived cell lines YU-1099 (harboring EGFR G719C/S768I mutation) and YU-1092 (carrying EGFR L861Q mutation). Furthermore, 10 mM osimertinib induced an EGFR phosphorylation inhibition in the YU-1099 cell line, and the inhibitory concentration for YU-1092 was between 30 and 100 nM. Notably the concentrations that inhibited phosphorylation were used to suppress the proliferation of both cell lines (20). An *in-vivo* study in the LC-F-29 EGFR G719A;S768I patient-derived xenograft (PDX) model found a significant shrinkage in tumor size ((>100) 45%, p<0.001 at day 14, versus the control group) (19). Tumor shrinkage was also detected in another *in-vivo* model, CTG-2453 NSCLC PDX model bearing the EGFR G719A;S768I mutation (19).

A few studies also showed the efficacy of osimertinib in NSCLC patients with uncommon mutations other than G719X, L861Q, and S768I. A study performed in Nanjing, China presented the first case of patients with novel and rare mutations 750_758del, I759S, and T751_I759delinsS, and durable clinical remission was achieved with the standard dose of osimertinib therapy (20). Notably, the patient had intractable terminal NSCLC but experienced a prolonged 52-week overall survival with Osimertinib (20). Another report involved two patients with p.I740_K745dupIPVAIK that showed a positive response to osimertinib therapy. After a few weeks of treatment, one patient’s brain nodule disappeared accompanied by a diminished left lung tumor; the other patient’s lung tumor shrank and brain metastasis disappeared (21). Two groups each reported the same novel but rare EGFR H773L alone or in combination with V774M that is sensitive to osimertinib. One study observed a reduced size of pulmonary nodule and pleural effusion up to 9 months; the other one reported more than 10-month PFS with osimertinib therapy (22, 23). Another case reported a patient carrying an uncommon L858R/D761Y compound mutation in EGFR. Osimertinib treatment lead to a PFS of 19 months and OS of more than 3 years (24).

## Different Responses of Uncommon EGFR Mutations to Osimertinib

The results of the previously published studies on uncommon mutations indicate these cases show varying response to osimertinib-centered therapy. A well-known osimertinib-resistant mutation is C797S, which interrupts the covalent bonding between osimertinib and mutant EGFR. This mutation was identified in samples from NSCLC patients (25). An *in-vitro* study showed that osimertinib fails to suppress the proliferation and phosphorylation of C797S and T790M mutation–positive NSCLC cells (25). A fair prevalence of C797S (6 out of 15) was found in in progressive T790M-positive NSCLC patients with acquired osimertinib resistance (25). In another investigation, 9 out of 13 samples maintaining T790M at the time of resistance carried EGFR C797S (26). The EGFR C797S mutation has been widely regarded as one of significant mechanisms inducing resistance of NSCLC to third-generation TKIs. One study reported the co-occurrence of C797S and T790M on the same allele (in cis) (25). A subsequent report further explored the allelic context of the two mutations and the effect on drug sensitivity (27). In 85% of the osimertinib-resistant MGH121 Res#1 cells, T790M and C797S were in cis, while the other 15% of cells had no T790M or C797S mutations on exon 20 alleles (27). Different allelic distribution of the two mutations (in trans) were also reported in one patient (25). The in-cis configuration caused a resistance to all three generation of EGFR-targeting TKIs alone or in combination (27). An engineered 293T cell line in which C797S and T790M were in trans demonstrated resistance to osimertinib, while treatment with combined first- and third-generation TKI therapy was efficacious (27).

In addition to C797S, some rare mutations including L792X, G796S, L718Q, G796R, G796D, and G724S play a role in inducing osimertinib resistance (28–38). L792X, G796S, and L718Q mutations account for 3%, 1%, and 1%, respectively, of all samples in the AURA3 and FLAURA studies (35, 36). Another study reported that osimertinib-resistant mutations in EGFR G796/C797, L792, and L718/G719 were identified in 24.7%, 10.8%, and 9.7% of osimertinib treatment–resistant individuals, respectively (37). The half inhibitory concentration (IC50) was significantly increased in L792X- and L718Q-positive cells compared with cells expressing the double mutations (Ex19del/T790M or L858R/T790M); L718Q conferred the strongest resistance to osimertinib when not coexisting with T790M (37).

Mutation of the G796 residue also appears after tumor progression upon osimertinib treatment (34, 38). The two case reports covering G796S/R and G796D highlighted the tumor heterogeneity and present novel mechanisms of osimertinib resistance (34, 38). Another rare driver mutation is G724S, which was detected in two patients with progressing NSCLC on osimertinib treatment (39). The emergence of this mutation is independent from T790M and it is sensitive to second-generation TKIs (30). Both *in-vitro* and *in-vivo* studies have confirmed the osimertinib-resistant effect induced by this mutation (30).

**Figure 1:**
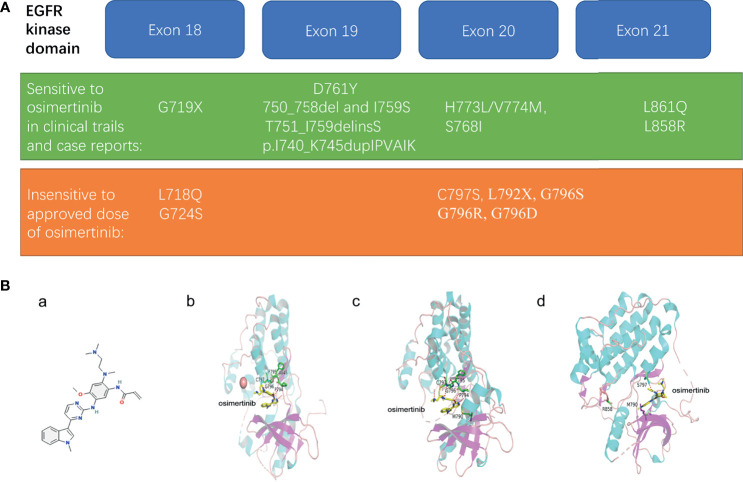
**(A)** Representative uncommon EGFR mutations that are sensitive or resistant to osimertinib treatment. **(B)** The structural modeling reveals the binding modes of sensitive or resistant mutations in complex with osimertinib. **(a)** structure of osimertinib (PubChem Compound Summary for CID 71496458, cited on 12/12/2021). Osimertinib is 4-(1-methylindol-3-yl)pyrimidin-2-amine in which one of the amino hydrogens is replaced by a 2-methoxy-4-[2-(dimethylamino)ethyl](methyl)amino-5 acrylamidophenyl group. **(b)** in silico modeling of the reversable binding mode of osimertinib in complex with wild type EGFR. **(c)** the binding simulations of the AZD9291-bound T790M EGFR mutant. **(d)** the strongly weakened binding of osimertinib to T790M/L858R/C797S EGFR mutant.

The sensitive and insensitive EGFR mutations to osimertinib are listed in **Figure 1A**.

## The Structural Elucidation Revealing Mechanisms of the Response Variation of Uncommon EGFR Mutations to Osimertinib

The structure of osimertinib was shown (**Figures 1B, a**). Previous studies have examined the selectivity of osimertinib binding to mutant EGFR using models by structural determination of crystallized molecules. One study showed a reversible binding of osimertinib to wild-type EGFR. Instead of forming a covalent bond through the interaction with cysteine residue at 797 (Cys797), the crystal structure of osimertinib binding wild-type EGFR revealed a reversible binding on the outer edge of the ATP-binding pocket (40). The ligand was sandwiched between EGFR’s P-loop and backbone between Pro794 and Cys797 (40). The relative stability of this binding came from osimertinib N4 hydrogen bonding to the hinge atom Met 793 N and osimertinib carbonyl oxygen hydrogen bonding to Cys 797 N (**Figures 1B, b**) (40).

After pinpointing the core structure involved in the binding, the group further confirmed that the decisive factor of osimertinib binding to EGFR was “the ability of the P-loop to form a closed or bent structure and also importantly, to contact the ligand through the side chain of the terminal P-loop Phe or Tyr residue” (40). The tip of the P-loop, “specifically residues Ser 720, Gly 721, Ala 722, Phe 723, and Gly 724, forming a stable and bent structure with the Phe723 side chain located underneath the P-loop” was the only change induced on osimertinib binding (40). This bent structure formed a 3.7 Å contact with osimertinib and could explain the selectivity for the EGFR kinase to some extent but not the entire binding profile observed in clinical practice (40).

When it came to the molecular events regarding osimertinib binding to specific mutant EGFR, however, the theories were conflicting. The Squire group held that “direct contact with either of the mutated amino acids, T790M or L858R, has no bearing on the AZD9291 binding mode.” Instead, these mutations only favored osimertinib binding by influencing protein conformation and molecular dynamics (40). In contrast, the model adopted by Yun group suggested that the flipped position of osimertinib (the phenyl moiety of the indole group pointing toward the gatekeeper residue) induced by the T790M mutation mediated an EGFR conformational change caused a stronger attractive van der Waals interactions between the indole and the Met 790 side chain, “as is evidenced by the shorter distance (∼3.8 Å versus ∼4.9 Å) and larger interaction surface,” which facilitated osimertinib binding (**Figures 1B, c**) (41).

The flexibility of osimertinib is responsible for drug sensitivity in some cases. The exon 20 insertion mutations in EGFR resulted in bending of the P-loop into the drug-binding site and narrowed the entrance, but osimertinib is flexible enough to pass through the entrance and dock at the drug binding site (42). In contrast, Lovly et al. reported a disruption of a stable bent P-loop conformation by the G724S mutation bound to Osimertinib (43). This bent P-loop conformation facilitated an energetically favorable contact between the F723 phenyl ring with the indole ring of osimertinib, contributing to the further covalent affinity (43). G724S rigidified the P-loop and displaced F723 from contact with Osimertinib (43).

In other cases, the mutant EGFR escaped from osimertinib binding through the loss of the covalent binding site or structural modification. C797S and G796S/R mutations abolish the covalent binding of osimertinib to EGFR (**Figures 1B, d**) (34, 37). A molecular simulation showed the greatest binding energy affinity for C797S complex signifying the weakest binding, compared with native EGFR and the L844V mutant (44). Secondary mutations in L792 and L718 residues prevented osimertinib from binding to EGFR by introducing a spatial confliction and decreased local hydrophobicity (37).

## The Osimertinib-Related Medical Progress Promotes the Therapy Targeting Uncommon EGFR Mutation- Positive NSCLC

The approval of osimertinib by the FDA enriched the current therapeutic regimes, improved progression-free survival and enhanced the overall survival of NSCLC patients. However, tumor escape from inhibition of targeted therapy has remained an issue. Several methods enabled precise application of osimertinib to maximize the efficacy.

**Figure 2:**
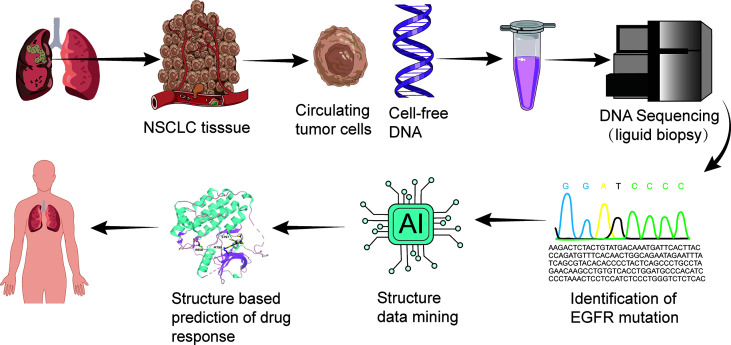
Liquid biopsy and structure-based drug response prediction promote osimertinib-centered precision medicine against uncommon EGFR mutation-positive NSCLC.

One method for the evaluation of candidate patients and directing towards precision medicine is liquid biopsy (**Figure 2**). Liquid biopsy profiles the genetic features of circulating carcinoma cells, which has advanced the understanding of precision medicine. Compared with traditional tissue biopsy, liquid biopsy has an easier method for sampling and enables faster identification of mutations that confer sensitivity or resistance to osimertinib treatment (45).

Another method combines machine learning and bioinformatical analysis on predicting response of uncommon EGFR mutations to Osimertinib (46) (**Figure 2**). Adopting this method, scientists presented four structural-function based uncommon EGFR mutation groups: classical like group, T790M-like group, Exon 20 loop insertion group, and P-loop alpha-C-helix compressing group, in order to guide clinical trial design and drug development with more precision (46).

Traditional pharmacological methods including *in-vivo* and *in-vitro* efficacy testing with biochemical methods may lead to the expansion of the therapeutic spectrum of osimertinib. One study reported that osimertinib is a lysine specific demethylase 1 inhibitor (47). Newly synthesized chemicals based on osimertinib exerted dual EGFR and histone deacetylase inhibitor activities (48). The new findings indicated that osimertinib treatment might lead to the remission of some unknown pathological types of cancerous disease.

To improve the efficacy of osimertinib, the drug may be administered at specific time points or in combination with different drugs. In one case report, a T790M-negative NSCLC patient with exon 19 deletion in EGFR (19del) was successfully rechallenged with osimertinib following acquired resistance to initial gefitinib and second-line Osimertinib (49). The newly designed and synthesized osimertinib derivatives and/or analogs answer the challenge of uncommon mutation mediated osimertinib resistance. A new sulfonyl-fluoride derivative of osimertinib forms a sulfonamide bond with the EGFR catalytic residue Lys745 and potently and irreversibly inhibits EGFR L858R/T790M/C797S (50).

## Conclusion and Perspectives

Osimertinib shows therapeutic potential, but its high price and low cost-effective, apart from drug resistance, are obstacles to its worldwide application. The seemingly promising clinical trial results are mixed with a possibility of cardiac toxicity from long-term osimertinib treatment (51). The creativity still lies in research and development. New drugs, such as EAI045, are in development that may ultimately improve upon osimertinib and improve patient treatment (52). Additionally, the usage of already available second generation TKIs provides another solution to identifying new treatments for NSCLC with acquired drug resistance (27, 53) Improvements in genome profiling technologies along with the artificial intelligence–assisted diagnosis and prescription promise a better usage of osimertinib.

## Author Contributions

CS wrote the manuscript. XY reviewed and wrote the manuscript. All authors contributed to the article and approved the submitted version.

## Conflict of Interest

The authors declare that the research was conducted in the absence of any commercial or financial relationships that could be construed as a potential conflict of interest.

## Publisher’s Note

All claims expressed in this article are solely those of the authors and do not necessarily represent those of their affiliated organizations, or those of the publisher, the editors and the reviewers. Any product that may be evaluated in this article, or claim that may be made by its manufacturer, is not guaranteed or endorsed by the publisher.
